# Medical Training of the Dental Student*Extracted from paper read before the Virginia State Dental Association, and published in full in the “Dental Cosmos,” April, 1922.

**Published:** 1922-06

**Authors:** Thomas B. Hartzell


### MEDICAL TRAINING OF THE DENTAL STUDENT*

BY THOMAS B. HARTZELL, M.D., D.M.D.

The charters of our colleges authorize their presidents to confer degrees upon their graduates on the completion of ordained courses of study. Therefore the individual who is graduated in dentistry is authorized to practice what he has been taught under the laws of his state and country. If during the course of his tutelage he has been instructed in medicine and surgery pertaining to his specialty, he has the legal right to apply his knowledge to the benefit of his patients.

I have long advocated the medical degree for the dentist, and the practice of dentistry as a specialty in medicine, and served as a member of the committee from the dental college of the University of Minnesota and the medical school of the University of Minnesota to plan a

* Extracted from paper read before the Virginia State Dental Association, and published in full in the "Dental Cosmos," April, 1922.

course, which, if adopted by our regents, would have permitted the colleges of medicine and dentistry to confer a degree in medicine and dentistry upon the student who satisfactorily completed such courses. Owing to the fact that it now costs the student seven years of study to secure a medical degree and become eligible to practice, and the further fact that the medical authorities were unwilling to concede to the lessening or modification of study to those working toward the end of a dental and medical degree, the best arrangement we could agree upon required nine years of college life, which to me seemed entirely out of proportion to the end sought. Therefore, no report was ever made by this committee.

Following this effort, I had the honor and enjoyed the privilege in association with the officers of the school of medicine, University of Minnesota, of arranging a course in medicine for dentists and dental students, which course comprised thirty lectures, covering in a comprehensive way the common interlocking diseases which the dentists and physicians most need to understand. I shall not at this time attempt to recount these courses of lectures. It is sufficient to say that they covered the subject of focal infection in its relation to general medicine, in its relation to obstetrics, in its relation to the eye, ear, nose, and throat, in its relation to surgery, in its relation to nerve and mental diseases, and in its relation to chronic diseases of the lungs, heart, and kidneys, pointing out to the student the important facts in the symptom complex of combined mouth infection and general disease.

In the teaching of this course, the heads of the departments in the medical school utilized the clinical material in our hospital to illustrate each subject in which instruction was given. The class gathered in a lecture room in the hospital, listened to the lecture illustrated, and was impressed by the case history and observation of the patient himself.

The first year in which this course was taught, we had thirty-five matriculants, the majority of whom were graduate dentists. The course continued to grow in favor until now a modification of it is being taught to the dental student. Such a course as this enables the student to make use of a systematic method of survey of any patient presenting symptoms of secondary disease who consults the dentist for dental service, or who consults the dentist with regard to a possible heart, joint, kidney, or other constitutional ailment which is alleged or believed to depend upon infection resident primarily in the oral tract.

It would indeed be an excellent thing if our medical and dental colleges could combine their teaching in such a manner that the medical and dental degrees could both be given in such a limit of time as would place it within the reach of the student.

One of the strong arguments for the course in medicine suggested by the foregoing remarks is that the present expense of dentistry to the average patient is becoming prohibitive by reason of the fact that we have too few dentists to serve the public. Also, it gives the dentist who was graduated without this medical training an apportunity to obtain such knowledge in the form of a postgraduate course. I am thoroughly convinced by the rearrangement of our courses, eliminating as I have suggested, certain subjects now taught in the dental school, such as physics, biology, chemistry, English, and technical drawing, which together require more than four hundred hours of the dental student's time, that this medical training could be given. The dental student should have the above subjects taught him in his preparatory years rather than in the dental course.

It must be remembered that to become a good dentist capable of the highest form of service the student must acquire a high degree of technical skill. He must in addition to his general knowledge of medicine and surgery have a greater degree of technical skill than any of the other specialties in medicine, and this can best be taught during the plastic stage of youth. The rewards for dental service do not justify nine years of school training to be taken out of the life of the individual for the sake of such a degree. We know that dentistry is an arduuous calling and we know from the statistics of the last ten years that the wastage from the dental profession has been equivalent to and in some years greater than the number of men graduated. That has been the case in the present year and will be true of the year 1922. Any effort on the part of the National Dental Association to increase the years of tutelage in dentistry must result, in the end, in defeating the object sought by such increase, namely, more and better dentists.

For this reason and the reasons already mentioned, the writer has advocated the elimination of certain non-essential subjects in the dental course and the concentration of teaching efforts on the technical phases of dentistry. The hours given to the less essential things in the present dental course should be devoted to acquiring a knowledge of clinical diseases, their underlying pathology and bacteriology, and the modern clinical treatment of them. The student having had the four-year course in dentistry involving bacteriology and pathology as at present taught and in addition to which we have supplied thirty weeks of ardent study of the important death-dealing diseases which more or less constantly confront the dentist, we would have created a type of man who can and will co-operate with the medical profession to a degree at the present time unheard of.

### EXAMINATION OF PATIENT

For a man who has received such training, the following scheme of survey of the patient has proven of the greatest value:

Name of the patient, sex, nationality, age, residence, present weight and usual weight, height, family history and occupation; for the female, the number of children and their ages, a statement of the patient's general complaint as fully recorded as the patient is able to give it. Then should come the family history—father, mother, brothers and sisters with their present state of health. If any are dead, the cause should be recorded. Then should come an examination of the circulatory system, a brief survey of the heart, arteries, pulse, blood pressure, temperature and blood-picture, which includes of course the hemoglobin, red cells and the number of white cells. A twenty-four-hour specimen of urine should be had and its examination should involve its specific gravity, test for albumin, sugar, casts, red blood cells, etc. A microscopic and chemical examination should be made of such urine.

The chemical examination, while in some measure superseded by the chemical examination of the blood, is still of great value to quickly get an idea of the condition of that most important avenue of elimination, the kidney. An individual who presents a urine with a normal specific gravity, without casts, albumin or sugar, is a case for any ordinary dental surgical interference; whereas, an individual, whose blood chemistry is such that sugar appears in the blood long before it makes its appearance in the urine, is certainly a case that requires the most careful and judicious attention. Coupled with this survey should be the physical examination of the mouth by the dentist, than whom there is no one so capable in the whole field of the healing art for this purpose.

This examination of the mouth and teeth themselves can be made both by the eye and digital examination. The color and general condition of the health of the membranes of the investing tissues of the teeth should be first noted. As you all know, if the investing tissues of the teeth and gums are normal, they are firm, pink, and the gingival tissue is closely attached to the teeth with no more than the normal depth of gingival crevice.

Tissues which vary from this normal appearance of health show the positive presence of infection by the mouth bacteria or "mouth molds," as I prefer to call them, evidence of which is reflected in the color of the tissue, the amount of edematous fluid contained in them and, as bacterial penetration increases, by a flow of pus under digital pressure from the gingival crevice in the gums. Many of the old chronic granulomata which have given no evidence of their presence may be detected by gentle pressure made in a circular movement over the root ends of such teeth, evincing in a few seconds, pain; whereas, such pressure over perfectly healthy root ends elicits no sense of tenderness in the periosteum overlying such areas.

In the further examination of the mouth of the patient, the quality of the saliva should be noted, the amount of putrefactive bacteria on the tongue should be observed, and the post-nasal space and tonsils examined. There is no one in the field of the healing art more competent to determine whether or not pus is present in the tonsils than the dentist, for his finger dexterity is highly developed. An ordinary dental mouth mirror placed between the pillars and below the tonsils with an index finger above the tonsil and gentle pressure applied, is the surest means of determining whether one has pus in the tonsils. Frequently, I have secured a gush of pus from a small, normally pink or submerged tonsil sufficient to cover the lens of an ordinary mouth mirror by this method.

Having completed the ocular and physical examination of the mouth and noted its results, one should in all cases have a complete radiographic examination made of the teeth and jaws, and after the films are made, they should be mounted in such relation to each other that when placed all together one can observe all the teeth in the maxillæ and mandible at a glance.

There is not a single thing indicated in this phase of general diagnostic procedure that the dentist can not with his present training do for himself, save the examination of the blood and urine. I find in different parts of the country men who have acquired the necessary training to do this for themselves also. I think of one such man now in the far west who maintains in his office, as I have in my own, complete facilities for blood examination and urinalysis. This indicates to my mind the trend in which dentistry is traveling, and while a survey, such as I have indicated to this point, is of inestimable value in forming an opinion of the general condition of the resistance of the patient, we can make it still more searching by following such a routine as that which I now recite.

After having gained the salient facts regarding the patient by the use of the above case survey, we may then take up a brief examination of the patient's head, eyes, sinuses, nose, throat, skin, lymph nodes, lungs, heart, genito-urinary organs, the gastro-intestinal tract, nervous and mental conditions, and the more important reflexes, including special questions about the appendix and the catamenia. From three to five questions under each one of these heads often serve to bring out the possible pathologic conditions in these fields. After having formed a reasonable estimate of the patient's present condition, one should in addition, under the head of habits and environment, inquire as to the appetite, bowel movements, sleep, exercise and fluid intake.

